# Preclinical Study of a Dual-Target Molecular Probe Labeled with ^68^Ga Targeting SSTR2 and FAP

**DOI:** 10.3390/ph17121647

**Published:** 2024-12-07

**Authors:** Huanhuan Liu, Xiaojun Zhang, Yue Pan, Jingfeng Zhang, Hui Wen, Cong Zhang, Xiaodan Xu, Guangyu Ma, Ruimin Wang, Jinming Zhang

**Affiliations:** Department of Nuclear Medicine, First Medical Center, Chinese PLA General Hospital, Fuxing Road 28, Beijing 100853, China

**Keywords:** somatostatin receptor 2, fibroblast activation protein, PET, heterodimer

## Abstract

Objective: Currently, ^68^Ga-labeled somatostatin analogs (SSAs) are the most commonly used imaging agents for patients with neuroendocrine tumors (NETs) in clinical practice, demonstrating good results in tumor diagnosis. For applications in peptide receptor radionuclide therapy (PRRT), targeted drugs should have high tumor uptake and prolonged tumor retention time. To enhance the uptake and retention of tracers in NETs, our goal is to design a ^68^Ga-labeled heterodimer for optimizing pharmacokinetics and assess whether this form is more efficacious than its monomeric equivalents. Methods: Using the somatostatin analog TATE and quinoline-based compound FAPI-46 as raw materials, we designed and synthesized ^68^Ga-labeled TATE-46. The labeling efficiency and stability were verified by Radio-HPLC. The receptor binding properties and tumor targeting were examined both in vitro and in vivo by using NCI-H727 (SSTR2/FAP, positive) and Mc38 (SSTR2/FAP, negative) cell lines and tumor-bearing mouse models. Preclinical evaluation was performed through cell uptake, pharmacokinetics, Micro PET, and biodistribution studies, and the results were compared with [^68^Ga]Ga-DOTA-TATE and [^68^Ga]Ga -FAPI-46. Immunohistochemistry and HE staining were performed on tumor tissues from tumor-bearing mice for further validation. Results: [^68^Ga]Ga-TATE-46 showed comparable SSTR2 and FAP targeting ability to monomeric TATE and FAPI-46 in cell uptake and PET imaging studies. [^68^Ga]Ga-TATE-46 exhibited significantly higher uptake in NCI-H727 (SSTR2/FAP, positive) tumors compared to [^68^Ga]Ga-DOTA-TATE (*p* < 0.001) and [^68^Ga]Ga-FAPI-46 (*p* < 0.001). No increased uptake of [^68^Ga]Ga-TATE-46 was observed in MC38 tumors (SSTR2/FAP, negative). Additionally, excess DOTA-TATE and/or unlabeled FAPI-46 significantly blocked the uptake of [^68^Ga]Ga-TATE-46 in NCI-H727 tumors (*p* < 0.001), confirming its dual-receptor targeting characteristics. The ex vivo biodistribution, immunofluorescence and immunohistochemistry results were in line with the in vivo imaging findings. Conclusion: Compared with ^68^Ga-labeled FAPI-46 and DOTA-TATE mono-specific tracers, the dual-target tracer [^68^Ga]Ga-TATE-46 improves tumor uptake, extends tumor retention, and enhances pharmacokinetics. It is an effective probe for non-invasive detection of tumors expressing FAP and SSTR2, and it is worth further studying its application in the expression of sstr2 and FAP-related tumors.

## 1. Introduction

Somatostatin receptors are overexpressed in a broad range of neuroendocrine tumor (NET) cells. To date, five human somatostatin receptor subtypes have been identified, including SSTR1-5. Among them, SSTR2 is the predominant subtype expressed in NETs, making it a popular target for somatostatin-based imaging and therapy [1]. Currently, three SSTR PET radiotracers are accessible: [^68^Ga]Ga-DOTA-TATE was approved by the U.S. Food and Drug Administration (FDA) in 2016; [^68^Ga]Ga-DOTA-TOC was approved by the European Medicines Agency (EMA) in 2016 and the FDA in 2019; and ⁶⁴Cu-DOTA-TATE was approved by the FDA in 2020. Although [^68^Ga]Ga-DOTA-NOC has not yet received approval from either the FDA or EMA, its application is approximately equivalent to that of [^68^Ga]Ga-DOTA-TOC and [^68^Ga]Ga-DOTA-TATE, showing similar accuracy in the detection of SSTR-positive diseases [2]. Recently, ¹⁷⁷Lu-DOTA-TATE (Luta Thera) received FDA approval. Despite its efficacy in neuroendocrine tumor patients, the therapy (patients were treated with a cumulative injected radioactivity dose ranging from 750 to 800 mCi (27.8–29.6 GBq), typically in four treatment cycles) is restricted by relatively low doses (23–29 Gy) delivered to the tumor because of the rapid clearance of the peptide from the bloodstream [3].

Fibroblast activation protein (FAP), a type II transmembrane serine protease, is highly expressed in cancer-associated fibroblasts (CAFs). It has a close connection with tumor growth, invasion, metastasis, immune suppression, and prognosis [4]. The expression of FAP in a wide variety of tumor types has made it a potential target for cancer imaging and radionuclide therapy in recent years [5]. Various quinoline-based FAP inhibitors, such as FAPI-02, FAPI-04, and FAPI-46 [6,7], have been developed in recent years. However, their short tumor retention time, while adequate for imaging, is not ideal for radionuclide therapy. Therefore, it is necessary to modify FAPI to enhance tumor targeting sensitivity and prolong tumor retention time in therapeutic applications [8].

In recent years, radiotracers targeting TATE or FAP have been extensively studied for tumor imaging, with some already in clinical use [9,10,11,12,13,14,15]. However, because of tumor heterogeneity and the intricate tumor–stroma interactions within multicellular systems, the diagnostic efficacy of typical mono-specific radiotracers is limited [16]. Therefore, the objective of this study is to create a dual-receptor-targeted peptide heterodimer probe that simultaneously targets SSTR2 and FAP, with the expectation of improving tumor detection sensitivity and extending tracer uptake and retention time. We evaluated the in vivo and in vitro performance of the developed tracer [^68^Ga]Ga-TATE-46 through a series of preclinical studies and compared it with the corresponding mono-targeted probes [^68^Ga]Ga-DOTA-TATE and [^68^Ga]Ga-FAPI-46.

## 2. Results

### 2.1. Chemistry and Radiochemistry

**Figure 1:**
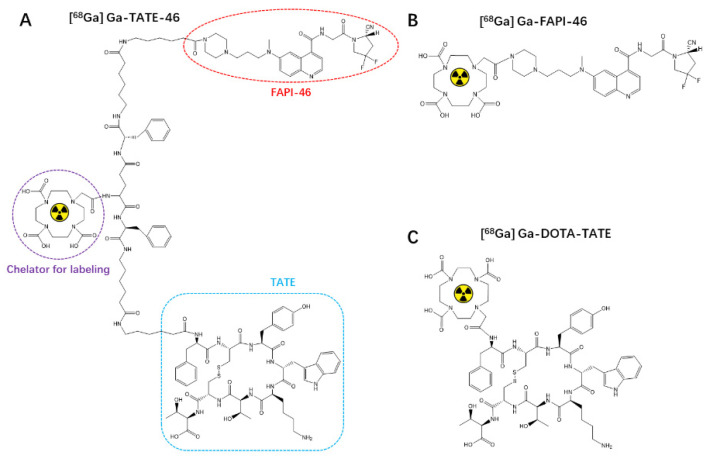
The chemical structures of [^68^Ga]Ga-TATE-46 (**A**), [^68^Ga]Ga-DOTA-TATE (**C**), and [^68^Ga]Ga-FAPI-46 (**B**).

**Figure 2:**
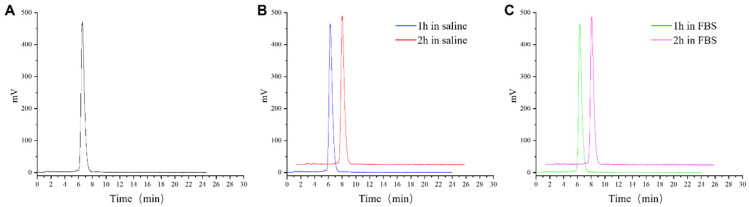
**(A**) Radiochemical HPLC analysis of [^68^Ga]Ga-TATE-46. (**B**) In vitro stability of [^68^Ga]Ga-TATE-46 in physiological saline over 1 and 2 h. (**C**) In vitro stability of [^68^Ga]Ga-TATE-46 in FBS over 1 and 2 h.

The chemical structure of synthesized TATE-46 is depicted in Figure 1. TATE-46 was characterized by MS and HPLC (Figure S1). Radiolabeling of ^68^Ga-TATE-46 was accomplished at an activity concentration of approximately 111 MBq/mL (molar activity, 103 GBq/μmol), and the radiochemical purity exceeded 95% after purification (Figure 2A). The radiochemical purity (RCP) of [^68^Ga]Ga-DOTA-TATE and [^68^Ga]Ga-FAPI-46 was greater than 99% as determined by radio-HPLC (Figure S2).

### 2.2. Stability and Log P of [^68^Ga]Ga-TATE-46

The Log *p* value of ^68^Ga-TATE-46 is −0.20 ± 0.05, while the Log *p* values of [^68^Ga]Ga-DOTA-TATE and [^68^Ga]Ga-FAPI-46 are −3.02 ± 0.08 and −3.05 ± 0.04, respectively. This indicates that [^68^Ga]Ga-TATE-46 has lower hydrophilicity compared to [^68^Ga]Ga-DOTA-TATE and [^68^Ga]Ga-FAPI-46. After incubating [^68^Ga]Ga-TATE-46 in physiological saline or FBS at 37 °C for 2 h, only one peak appeared on the HPLC chromatogram, indicating that it is stable in both in vitro systems and remains stable during the testing period (Figure 2).

### 2.3. In Vitro Cell Assays and Pharmacokinetics

**Figure 3:**
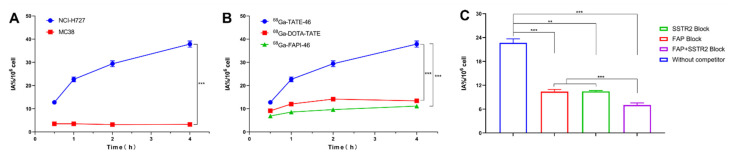
In vitro studies. (**A**) Uptake of [^68^Ga]Ga-TATE-46 by NCI-H727 and MC38 cells at 30 min, 1 h, 2 h, and 4 h. (**B**) Uptake of [^68^Ga]Ga-TATE-46, [^68^Ga]Ga-DOTA-TATE, and [^68^Ga]Ga-FAPI-46 by NCI-H727 cells at 30 min, 1 h, 2 h, and 4 h. (**C**) Blocking of [^68^Ga]Ga-TATE-46 uptake by NCI-H727 cells with unlabeled DOTA-TATE, FAPI-46, and TATE + FAPI-46 (data are expressed as mean ± SD, *n* = 3; ** *p* < 0.01, *** *p* < 0.001).

Cell uptake studies using NCI-H727 cells were conducted with [^68^Ga]Ga-TATE-46, [^68^Ga]Ga-DOTA-TATE, and [^68^Ga]Ga-FAPI-46. All tracers showed increased uptake with extended incubation times (Figure 3B). At all of the time points investigated, the cellular uptake of [^68^Ga]Ga-TATE-46 in NCI-H727 cells was significantly higher than that of [^68^Ga]Ga-DOTA-TATE and [^68^Ga]Ga-FAPI-46 (all *p* < 0.001). The targeting specificity of [^68^Ga]Ga-TATE-46 was assessed through cell blocking experiments (Figure 3C). Co-incubation with unlabeled TATE, FAPI-46, or TATE + FAPI-46 at 1000 times the molar amount of [^68^Ga]Ga-TATE-46 resulted in a significant reduction in the cellular uptake of [^68^Ga]Ga-TATE-46 in NCI-H727 cells (all *p* < 0.01). In the negative control MC38 cells, the cellular uptake of [^68^Ga]Ga-TATE-46 was also significantly lower at all time points compared to NCI-H727 cells (all *p* < 0.001, Figure 3A).

**Figure 4:**
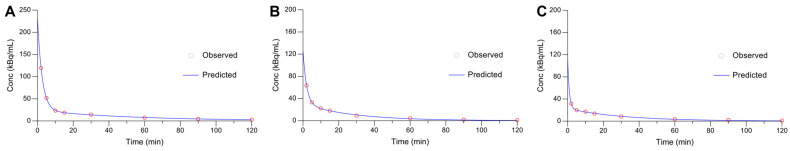
Time–activity curves for [^68^Ga]Ga-TATE-46 (**A**), [^68^Ga]Ga-DOTA-TATE (**B**), and [^68^Ga]Ga-FAPI-46 (**C**) in BALB/C mice, respectively.

The pharmacokinetic curves in BALB/c mice confirmed that the pharmacokinetics of [^68^Ga]Ga-TATE-46, [^68^Ga]Ga-DOTA-TATE, and [^68^Ga]Ga-FAPI-46 follow a two-compartment model. The distribution half-life and elimination half-life of [^68^Ga]Ga-TATE-46 were 1.79 min and 37.75 min, respectively (Figure 4A), while the corresponding values for [^68^Ga]Ga-DOTA-TATE and [^68^Ga]Ga-FAPI-46 were 1.38 min and 19.50 min (Figure 4B) and 0.64 min and 21.77 min (Figure 4C), respectively.

### 2.4. Biodistribution 

**Figure 5:**
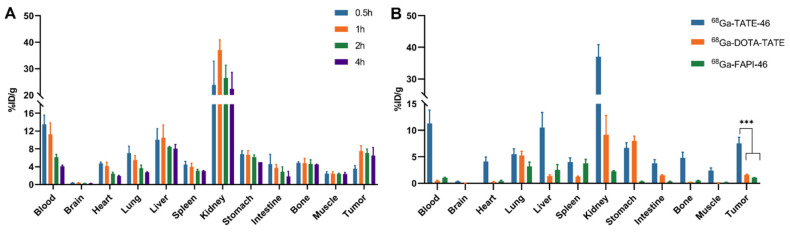
(**A**) Biodistribution of [^68^Ga]Ga-TATE-46 in NCI-H727 tumor-bearing mice at 0.5, 1, 2, and 4 h post-injection (each mouse received 5.55–7.4 MBq). (**B**) Biodistribution of [^68^Ga]Ga-TATE-46, [^68^Ga]Ga-DOTA-TATE, and [^68^Ga]Ga-FAPI-46 in NCI-H727 tumor-bearing mice at 1h p.i. (*** *p* < 0.001).

To further evaluate the pharmacokinetic properties of [^68^Ga]Ga-TATE-46 in vivo, we investigated the distribution of the three tracers in tumor-bearing mice through biodistribution studies. As shown in Figure 5A and Table S1, at 0.5, 1, 2, and 4 h post-injection (p.i.), the tumor uptake of [^68^Ga]Ga-TATE-46 was 3.56 ± 0.69, 7.53 ± 1.13, 7.08 ± 0.88, and 6.48 ± 1.82% ID/g, respectively, demonstrating good uptake and retention in the tumor, consistent with the PET imaging results. In normal organs, [^68^Ga]Ga-TATE-46 showed higher uptake in the liver and kidneys, but the uptake gradually decreased over time, indicating that the tracer is primarily excreted through the liver and kidneys, which is similar to the PET imaging results. For comparison, we studied the biodistribution of [^68^Ga]Ga-DOTA-TATE and [^68^Ga]Ga-FAPI-46 in NCI-H727 tumor-bearing mice at 1 h p.i. (Figure 5B and Table S2). The tumor uptake of [^68^Ga]Ga-DOTA-TATE was 1.57 ± 0.17% ID/g, and that of [^68^Ga]Ga-FAPI-46 was 1.07 ± 0.01% ID/g, both lower than [^68^Ga]Ga-TATE-46 (*p* < 0.001).

### 2.5. PET Imaging

**Figure 6:**
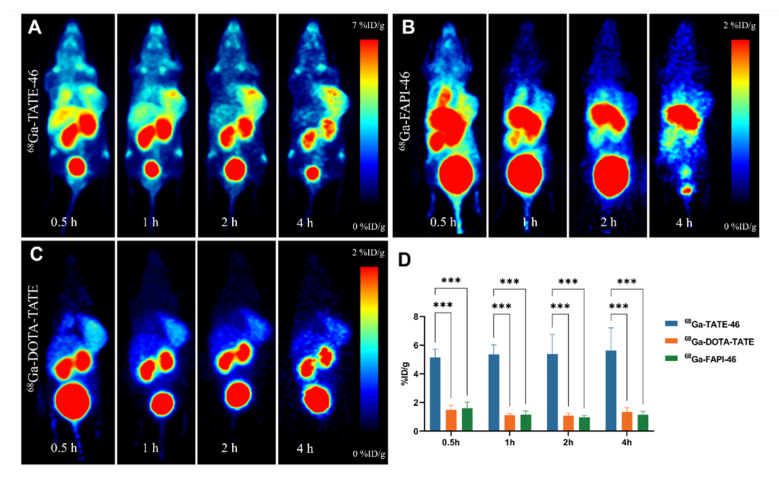
Micro PET scans of NCI-H727 tumor-bearing mice with [^68^Ga]Ga-TATE-46. (**A**) [^68^Ga]Ga-TATE-46, (**B**) [^68^Ga]Ga-FAPI-46, and (**C**) [^68^Ga]Ga-DOTA-TATE (each injected with 5.55–7.4 MBq) at 30 min, 1 h, 2 h, and 4 h post-injection. (**D**) Quantitative analysis of tumor uptake from PET images using ROIs, *n* = 3; ***p < 0.001.

To make a comparison of the in vivo targeting efficiency between single-targeted and dual-targeted tracers, we used PET/CT imaging in NCI-H727 tumor-bearing nude mice. PET images were obtained at 30 min, 1 h, 2 h, and 4 h post-injection of the tracers. As shown in Figure 6A, [^68^Ga]Ga-TATE-46 is primarily excreted through the liver, gastrointestinal tract, and kidneys. At 30 min post-injection, the NCI-H727 tumors are clearly visible, and the tumor imaging remains clear at 1, 2, and 4 h. The metabolism of [^68^Ga]Ga-FAPI-46 is similar to that of [^68^Ga]Ga-TATE-46, with a high accumulation of radioactivity in the liver, gastrointestinal tract, and kidneys. Despite the high radioactive distribution throughout the body, the tumors are clearly visible on PET images (Figure 6B). [^68^Ga]Ga-DOTA-TATE shows high radioactive accumulation in the bladder and kidneys (Figure 6C). After injection of [^68^Ga]Ga-DOTA-TATE, the tumors are clearly visible early on but become gradually blurred over time. Quantitative analysis of tracer uptake in the tumors was performed by drawing regions of interest (ROIs) on the PET images, with the %ID/g of the three tracers in NCI-H727 tumors shown in Figure 6D. The tumor uptake of [^68^Ga]Ga-TATE-46 is significantly higher than that of [^68^Ga]Ga-FAPI-46 and [^68^Ga]Ga-DOTA-TATE.

**Figure 7:**
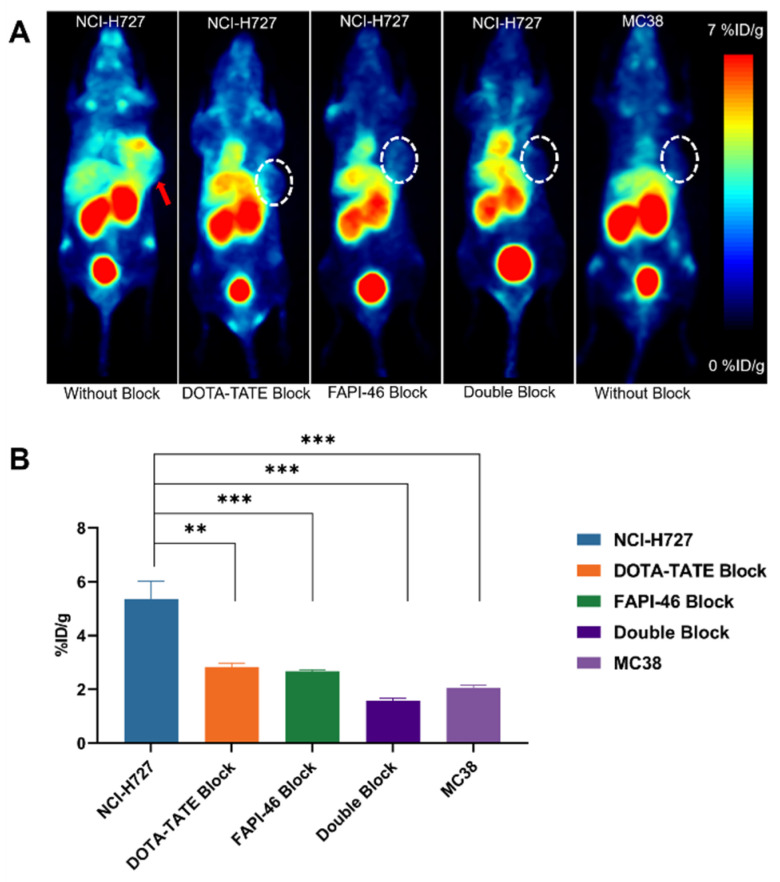
Micro PET imaging and quantitative analysis demonstrate the specificity of [^68^Ga]Ga-TATE-46 for SSTR2 and FAP expression in tumors. (**A**) PET images of NCI-H727 and MC38 tumor-bearing mice at 1 h post-injection of [^68^Ga]Ga-TATE-46 (5.55–7.4 MBq) with blocking agents DOTA-TATE (10 μg/g), FAPI-46 (10 μg/g), or DOTA-TATE (10 μg/g) + FAPI-46 (10 μg/g). The red arrows are tumors. (**B**) ROI quantitative analysis of tumor uptake from PET images. (** *p* < 0.01, *** *p* < 0.001).

Evaluation of the in vivo binding specificity of [^68^Ga]Ga-TATE-46 to SSTR2 and FAP through multiple blocking experiments. As shown in Figure 7A, when [^68^Ga]Ga-TATE-46 was co-injected with blocking agents DOTA-TATE (10 μg/g), FAPI-46 (10 μg/g), or DOTA-TATE (10 μg/g) + FAPI-46 (10 μg/g), the tumor uptake of [^68^Ga]Ga-TATE-46 was significantly reduced (all *p* < 0.001). Notably, the tumor images in the dual-blocking group were much more blurred compared to the single-blocking groups.

For control purposes, the in vivo performance of [^68^Ga]Ga-TATE-46 was investigated in MC38 tumor-bearing mice, which exhibit low expression of SSTR2 and FAP (Figure 7A). Very low uptake of [^68^Ga]Ga-TATE-46 was noticed in MC38 tumors, comparable to that in the dual-blocking group. The ROI quantitative analysis of MC38 tumors is shown in Figure 7B. At 1 h, the uptake of [^68^Ga]Ga-TATE-46 in MC38 tumors was 2.05 ± 0.09% ID/g (*p* < 0.001), significantly lower than that observed in NCI-H727 tumors.

### 2.6. Immunostaining and HE

**Figure 8:**
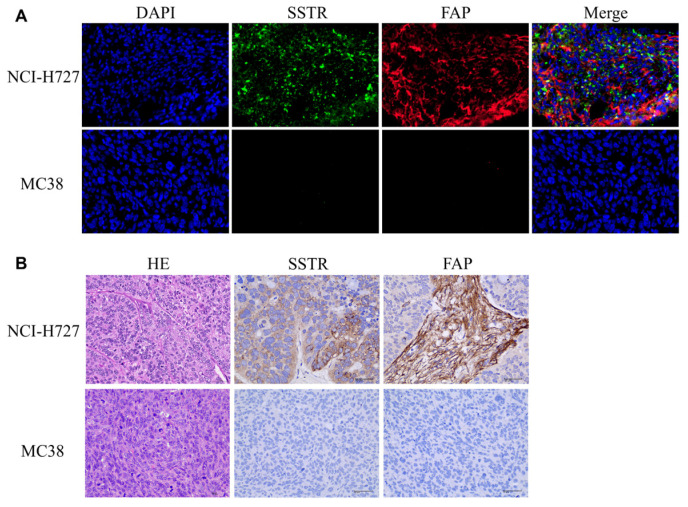
Immunofluorescence (**A**) and immunohistochemistry/HE staining (**B**) analyses of SSTR2 and FAP in NCI-H727 and MC38 tumors.

Immunohistochemical (IHC) staining analysis of SSTR2 and FAP expression in tumor tissues. As shown in Figure 8B, NCI-H727 tumors exhibit high expression of SSTR2 and FAP, while MC38 tumors show low or absent expression of SSTR2 and FAP proteins. Immunofluorescence confirms that the expression of SSTR2 and FAP proteins is higher in NCI-H727 cells compared to the negative control cell model, MC38 (Figure 8A).

## 3. Discussion

Despite the successful clinical outcomes of radiotracers targeting SSTR2 or FAP, single-receptor recognition may limit their sensitivity for disease detection, especially in early stages, due to tumor heterogeneity and relatively low receptor expression density [17]. To overcome this issue, various dual-targeting molecular probes that bind to different receptors have emerged over the past decade [8,16,18,19,20,21,22,23,24]. These dual-targeting probes can bind simultaneously or separately to different target structures on tumor cells, such as FAPI-RGD that binds both FAP and integrin αvβ3, prostate cancer dual-target probes for prostate-specific membrane antigen (PSMA) and GPPR, and FAPI-LM3 targeting both SSTR2 and FAP. These dual-targeting probes leverage the synergistic targeting ability of two ligands, enhancing cancer visualization [5]. For example, [^68^Ga]Ga-FAPI-RGD and [^68^Ga]Ga-FAPI-LM3 have both undergone preliminary clinical application with promising results.

The increasing interest in the development of dual-targeting radiopharmaceuticals is due to their significant improvement in tumor targeting compared to single-target probes. Dual-targeting probes can bind to two different target structures, thus exhibiting markedly increased target cell affinity and specificity. Furthermore, the use of radiolabeled heterodimers can address the common issue of limited tumor visualization sensitivity due to receptor expression differences among various tumor lesions [25]. However, the heterodimer strategy proves to be a double-edged sword in the development of radiopharmaceuticals. It might boost the efficacy of tumor detection, yet it could also raise the risk of false positives. The development of heterodimer radiotracers may offer a way to optimize the structure of radiotracers used in tumor diagnosis, avoiding the need to combine each single-agent radiotracer [22]. Previously, homodimer radiotracers have been shown to improve tumor uptake and retention [26,27]. Additionally, the development of heterodimer radiotracers targeting multiple biological receptors can improve tumor targeting efficacy and reduce patient discomfort, radiation burden, and treatment costs compared to using each single-agent radiotracer in the same patient [22]. Thus, developing radiotracers that bind to multiple receptors is crucial as they can be used for imaging whenever any receptor is expressed on the tumor, thereby improving detection sensitivity [16].

With ^68^Ga-DOTA-TATE and ^177^Lu-DOTA-TATE approved by the FDA, the development of somatostatin (SS) peptide analogistic for the detection and treatment of neuroendocrine tumors has been fruitful, and fibroblast-activating protein-alpha (FAP) targeting radiolands has recently shown high diagnostic potential. However, whether targeting SSTR or FAP, the blood clearance of these structures is rapid, which limits their absorption in the tumor, resulting in their therapeutic value being compromised by the short tumor residence time. In 2019, Anastasia [6] et al. published the development of activated protein targeting radioactive tracers to improve tumor retention, and concluded that the tracer FAPI-46 could significantly prolong the tumor accumulation time. In addition, radiolabeled SSTR activators are more likely than antagonists to internalize into tumor cells both in vitro and in vivo, resulting in the accumulation of radioactivity in tumor cells [28]. Therefore, in order to improve the uptake and retention time of tracers in the tumor, we took advantage of the favorable properties of heterodimers. In order to reduce the rate of clearance of the tracer in the blood, we also added Phe to increase the lipid solubility of the ligand, and finally designed and synthesized a double-specific dimer radiotracer targeting SSTR2 and FAP for cancer imaging and even future therapeutic studies. [^68^Ga]Ga-TATE-46 offers advantages such as ease of labeling and good stability, effectively binding to the target. Biodistribution studies show that [^68^Ga]Ga-TATE-46 accumulates effectively in the tumor region; the data indicated that it had a lower tumor/muscle ratio than the reported ^68^Ga-FAPI-LM3 (3–4 vs. 4–5), and [^68^Ga]Ga-TATE-46 was similar to ^68^Ga-FAPI-LM3 [5]; strong physiological uptake was observed in blood, liver, kidney and spleen. In addition, the uptake of [^68^Ga]Ga-TATE-46 in bone and muscle is also high, which poses a challenge to the clinical application of tracers. Therefore, we consider further optimizing the structure of the probe to reduce the uptake of the probe in non-target organs.

[^68^Ga]Ga-TATE-46 exhibits comparable SSTR2 and FAP targeting ability to its monomeric counterparts, TATE and FAPI-46, in cell uptake and PET imaging studies. [^68^Ga]Ga-TATE-46 shows high uptake in SSTR2/FAP-positive NCI-H727 tumors, significantly higher than [^68^Ga]Ga-DOTA-TATE (*p* < 0.001) and [^68^Ga]Ga-FAPI-46 (*p* < 0.001). No increased uptake of [^68^Ga]Ga-TATE-46 was observed in MC38 tumors (SSTR2/FAP, negative). Additionally, excess DOTA-TATE and/or unlabeled FAPI-46 significantly blocked the uptake of [^68^Ga]Ga-TATE-46 in NCI-H727 cells (*p* < 0.001), confirming its dual-receptor targeting specificity. The ex vivo biodistribution and immunofluorescence, immunohistochemistry results are consistent with the in vivo imaging findings. Compared to single-specificity tracers, the dual-receptor targeting strategy enhances tumor targeting efficiency in NCI-H727 tumors and prolongs the tumor retention time, indicating that [^68^Ga]Ga-TATE-46 is a promising non-invasive tracer for detecting tumors that simultaneously express SSTR2and FAP (e.g., nasopharyngeal carcinoma, thyroid carcinoma and breast cancer), with good potential for clinical translation. In addition, in order to further improve the efficiency of PRRT treatment and reduce toxic and side effects, it is necessary to prolong the half-life of the targeted molecule and increase its uptake and retention time in the tumor. From this perspective, our research can be said to be successful. However, in reducing the uptake in the kidney and reducing the toxic side effects, our research did not make TATE-46 a suitable candidate for therapeutic applications. Its high renal and bone uptake blocked its potential therapeutic applications. Therefore, our follow-up research direction focused on the structural modification of TATE-46. Once the imaging radionuclide ^68^Ga is replaced by therapeutic radionuclides (such as ^177^Lu or ^225^Ac), we hope that it can also provide a better therapeutic effect than ^177^Lu-DOTA-TATE.

## 4. Material and Methods

### 4.1. General Materials

All the chemicals, solvents, and reagents (of analytical grade) utilized for synthesis and analysis were procured from Macklin Biochemical Technology Co., Ltd. (located in Shanghai, China). The ^68^Ge/^68^Ga generator (ITM Medical Isotopes GMBH, Munich, Germany) was used on-site to elute ^68^Ga. Radioactivity was measured using the CRC-25R dose calibrator (Capintec, Inc., Ramsey, NJ, USA) and the γ-Counter (AMG 425-6c1, Hidex, Turku, Finland). The radiochemical labeling efficiency and radiochemical purity were tested using the Radio-TLC scanner (Eckert & Ziegler Eurotope GmbH, Berlin, Germany) and high-performance liquid chromatography (HPLC, Waters, Milford, MA, USA). Analysis column Phenomenex Gemini 5 μm 100A C18 (4.6 mm × 150 mm). Small-animal PET scans were conducted using the Super Nova PET/CT scanner (Super Nova PET/CT, Ping Sheng Medical Technology Co., Ltd. Kunshan City, Jiangsu Province, China).

### 4.2. Chemical Synthesis, Radiolabeling and Quality Control

A detailed schematic demonstration of chemical synthesis procedures is elaborated in the Supplementary Materials. During the labeling process, the generator was rinsed with 2 mL of 0.05 M HCl, and the waste solution was discarded. Then, 2 mL of 0.05 M HCl was used to elute ^68^Ga into the reaction vessel, which had 20 µg of precursor dissolved in 30 µL of high-purity water and 130 µL of 1 M sodium acetate already added. The reaction vessel was then sealed and heated to 85 °C for 10 min. The radiochemical purity was analyzed using Radio-HPLC. If the purity was over 95%, the product was directly neutralized and passed through a 0.22 μm sterile filter. If the purity was below 95%, the mixture was permitted to cool down to room temperature, then 10 mL of injection water was added, mixed, and diluted. The labeled mixture was passed through a Vac C-18 column, and the column was washed with 10 mL of pure water to elute free ^68^GaCl_3_. The radiolabeled product was eluted with 0.5 mL of ethanol, diluted with 5 mL of injection water, and filtered through a 0.22 μm sterile filter to obtain the final injectable solution for subsequent experiments. The radiochemical purity of the radiopharmaceutical was determined using Radio-HPLC with a mobile system of water containing 0.4% phosphoric acid (A), and acetonitrile (B) was used at a flow rate of 1 mL/min. This was conducted according to the following gradient program: constant 5% B and 95% A solution for 3 min, then going from 5% to 25% B in 10 min, at 25% for 5 min.

### 4.3. In Vitro Stability and Partition Coefficient

A measure of 5 µL of [^68^Ga]Ga-TATE-46 (1.85 MBq) was added to 300 µL of PBS or FBS and incubated at 37 °C for 2 h. Subsequently, the radiochemical purity was determined using HPLC.

In a 15 mL centrifuge tube, 2.9 mL of PBS (0.1 mol/L, pH = 7.4) and 3 mL of n-octanol were added. [^68^Ga]Ga-TATE-46, [^68^Ga]Ga-DOTA-TATE, or [^68^Ga]Ga-FAPI-46 (14.8 MBq, 100 µL) were added to each tube. The mixture was vortexed at room temperature for 2 min to ensure thorough blending, and then centrifuged at room temperature (effective centrifuge radius 12 cm, 3000 rpm) for 5 min. After allowing sufficient time for phase separation, three 500 µL samples were taken from each layer. The radioactivity of each sample was measured using a γ-counter (count per minute, CPM), and log *p* = log (n-octanol CPM/water CPM) was calculated. The experiment was repeated three times.

### 4.4. Cell Lines and Animal Models

The NCI-H727 and Mc38 cell lines were supplied by the Stem Cell Bank of the Chinese Academy of Sciences and were cultivated in Roswell Park Memorial Institute (RPMI-1640) medium with 10% (*v*/*v*) fetal bovine serum (FBS) at 37 °C with 5% CO2. All cells were cultured until they reached 80–90% confluence before undergoing trypsinization.

Female BALB/c nude mice were obtained from Charles River Laboratories (Beijing, China). The mice were approximately 3–4 weeks old and had a weight of 13–15 g. Approximately 5 × 10^6^ cells were implanted into the right shoulder of each mouse to establish an NCI-H727 tumor model and Mc38 tumor model. Once the tumor volume reached 200–300 mm^3^, the mice were used for imaging or biodistribution studies. All animal experiments were carried out in accordance with the National Institutes of Health Guidelines for the Care and Use of Laboratory Animals and were approved by the Animal Protection and Utilization Committee of the General Hospital of the PLA (Approval No.: S2023-209-1).

### 4.5. In Vitro Cellular Experiments

NCI-H727 cells were placed in a 24-well plate and cultivated for 24 h. The original medium was removed, and fresh RPMI-1640 was added. Then, 100 µL of [^68^Ga]Ga-TATE-46, [^68^Ga]Ga-DOTA-TATE, or [^68^Ga]Ga-FAPI-46 (1.48×10^4^ Bq) was added to each well, and the cells were incubated for 30 min, 1 h, 2 h, and 4 h (with three replicates for each time point). The culture medium was taken away, and the cells were washed twice with cold PBS (pH 7.4). Then, the cells were lysed using 0.5 mL of 1 M NaOH.

In blocking experiments, an excessive amount of unlabeled FAPI-46, DOTA-TATE, or FAPI-46 + DOTA-TATE (10 µg/well) was added as an inhibitor along with [^68^Ga]Ga-TATE-46 to the cells. After 1 h, the cells were processed using the same method. Finally, the cell lysates were gathered, and the radioactivity was measured using a γ-counter. Mc38 cells were used as a negative control, with only [^68^Ga]Ga-TATE-46 (1.48 × 10^4^ Bq/well, 100 µL) added, and the rest of the procedure was followed as described above.

### 4.6. Pharmacokinetics Studies

Nine female healthy BALB/c mice were randomly divided into three groups (with three mice in each group) using a random number table. Each group was administered 100 µL of [^68^Ga]Ga-TATE-46, [^68^Ga]Ga-DOTA-TATE, or [^68^Ga]Ga-FAPI-46 (0.37 MBq) via tail vein injection. Blood samples (5 µL × 3) were collected at 2, 5, 10, 15, 30, 60, 90, and 120 min post-injection via tail vein. The radioactivity in the blood was measured with a γ-counter and converted to blood radioactivity concentration (kBq/mL). The pharmacokinetic data (time–blood radioactivity concentration) were analyzed by using the two-compartment model in the pharmacokinetic software WinNonlin (Certara, Louis, MO, USA, 2018-06) to determine the distribution and clearance half-life parameters of the radiopharmaceuticals in the mice.

### 4.7. Biodistribution Studies

The NCI-H727 tumor-bearing mice were randomly assigned to four groups (with three mice in each group) and were injected with [^68^Ga]Ga-TATE-46 (1.48 MBq, 100 µL). The mice were put to death at 0.5, 1, 2, and 4 h post-injection (p.i.). The tissues and organs of concern were gathered, weighed, and measured for radioactivity using a γ-counter. Additionally, [^68^Ga]Ga-DOTA-TATE and [^68^Ga]Ga-FAPI-46 were used as control groups in the biodistribution study of the NCI-H727 tumor model at 1 h p.i. After performing time decay correction, the radiotracer uptake in each tissue and organ was calculated as %ID/g.

### 4.8. Small-Animal PET Imaging

Each set of NCI-H727 tumor xenograft models (n = 3) was administered with [^68^Ga]Ga-TATE-46, [^68^Ga]Ga-DOTA-TATE, or [^68^Ga]Ga-FAPI-46 (5.55–7.4 MBq, 150 µL/mouse) through the tail vein. Static PET scans were performed at 30 min, 1 h, 2 h, and 4 h after injection for 10 min each. In blocking studies, unlabeled precursors (FAPI-46, DOTA-TATE, FAPI-46 + DOTA-TATE, 10 mg/kg) were co-injected with [^68^Ga]Ga-TATE-46. After image acquisition, the images were reconstructed using the three-dimensional ordered subset expectation maximization (3D-OSEM) algorithm and analyzed on the Recon/Avator-s-10 workstation. The percentage of injected dose per gram of tissue (%ID/g) was determined by delineating regions of interest.

### 4.9. Immunohistochemical and Hematoxylin–Eosin Staining

NCI-H727 and Mc38 tumor tissues were collected and fixed in 4% formaldehyde. Paraffin sections were prepared using immunohistochemistry techniques and deparaffinized through an alcohol gradient. After antigen retrieval, endogenous peroxidases were blocked, and the tissue sections were treated with 3% bovine serum albumin (BSA) to minimize non-specific binding. Primary antibodies were incubated at 4 °C throughout the night, followed by incubation with secondary antibodies at room temperature for 30 min. The antibodies were diluted to the required concentrations according to the manufacturer’s instructions. After DAB staining, nuclear staining was observed, and the sections were dehydrated, mounted, and examined under a microscope. After deparaffinization, the sections were stained with hematoxylin and eosin and dehydrated for pathological analysis.

### 4.10. Statistical Analysis

Statistical analysis was carried out by means of SPSS 26.0 (New York, NY, USA, 2019-05) and GraphPad Prism 8.0 (San Diego, CA, USA). Statistically, a *p*-value lower than 0.05 was regarded to indicate a significant difference. For the data that conformed to a normal distribution, the mean ± standard deviation was employed to represent the data. For non-normally distributed data, the median and interquartile range were utilized to describe dispersion. Independent sample t-tests were implemented for normally distributed data, and tests for homogeneity of variance were also performed. For data that did not meet the normal distribution assumption, the Wilcoxon rank-sum test was adopted.

## 5. Conclusions

This study successfully developed a peptide heterodimer tracer, [^68^Ga]Ga-TATE-46, that simultaneously targets FAP and SSTR2. This tracer demonstrates excellent in vitro and in vivo performance. Compared to single-specificity tracers like ^68^Ga-labeled FAPI-46 and DOTA-TATE, the dual-targeting tracer enhances tumor uptake, prolongs tumor retention, and improves pharmacokinetics. However, further structural modifications to [^68^Ga]Ga-TATE-46 are needed to accelerate the clearance from normal tissues. Therefore, [^68^Ga]Ga-TATE-46 is an effective non-invasive probe for detecting tumors that simultaneously express SSTR2 and FAP, and it is expected to be used for clinical diagnosis and subsequent radionuclide targeted therapy research. In addition, the targeting strategy in this study is expected to contribute to a broader discussion.

## Data Availability

All data can be found in manuscripts and Supplementary Materials.

## Supplementary Materials

The following supporting information can be downloaded at https://www.mdpi.com/article/10.3390/ph17121647/s1: Figure S1: Chemical Synthesis and Mass spectra of TATE-46; Figure S2: Analytic radio-HPLC chromatograms of ^68^Ga-DOTA-TATE (A) and ^68^Ga-FAPI-46 (B); Table S1: Biodistribution results of ^68^Ga-TATE-46 in NCI-H727 tumor-bearing mice at 0.5, 1, 2, and 4 hours p.i.; Table S2: Biodistribution of ^68^Ga-TATE-46, ^68^Ga-DOTA-TATE, and ^68^Ga-FAPI-46 in NCI-H727 tumor-bearing mice at 1h p.i.
