# Supplementary material for: Preclinical Study of a Dual-Target Molecular Probe Labeled with 68Ga Targeting SSTR2 and FAP
Source: Pharmaceuticals (Basel). 2024 Dec 7;17(12):1647. doi: 10.3390/ph17121647 (PMC11677724; doi:10.3390/ph17121647)
Supplement: Supplementary file 1 [file pharmaceuticals-17-01647-s001.zip › pharmaceuticals-3347857-supplementary.pdf]

Supplementary materials

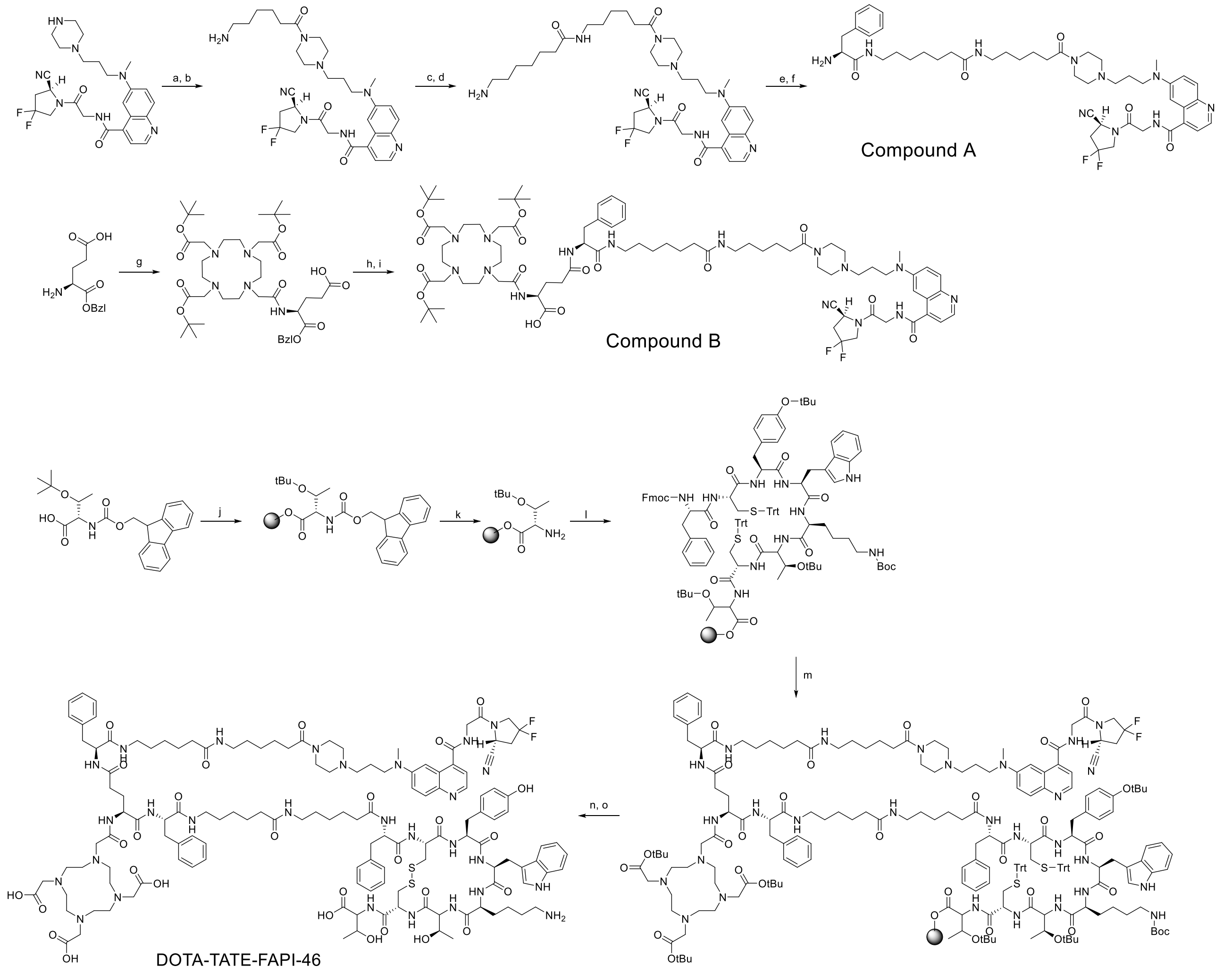

(a)N-Benzyloxycarbonyl-6-aminohexanoic acid, HBTU, DIPEA, DMF, r.t., (b) MeOH, Pd/C, H2, r.t., (c) N-Benzyloxycarbonyl-6-aminohexanoic acid, HBTU, DIPEA, DMF, r.t., (d) MeOH, Pd/C, H2, r.t., (e) CBZ-L-phenylalanine, HBTU, DIPEA, DMF, r.t., (f) MeOH, Pd/C, H2, r.t., (g) DOTA-tris(tBu)ester NHS ester, triethylamine, DCM, r.t. 24h, (h) Compound A, HBTU, DIPEA, DMF, r.t., (i) MeOH, Pd/C, H2, r.t., (j) 2-CTC-Resin, DCM, (k) 20% piperidine in DMF; (l) coupling step used for amino acids (HBTU, DIPEA in DMF), including Fmoc-Cys(Trt)-OH, Fmoc-Thr(tBu)-OH, Fmoc-Lys(Boc)-OH, Fmoc-Trp(Boc)-OH, Fmoc-Tyr(tBu)-OH, Fmoc-Cys(Trt)-OH, Fmoc-Phe-OH. Fmoc deprotection is carried out in 20% pyridine in DMF; (m) coupling step used for amino acids, linker groups, or chelators (HBTU, DIPEA in DMF), including Fmoc-6-Aminohexanoic Acid, Fmoc-6-Aminohexanoic Acid, Fmoc-Phe-OH, Compound B. Fmoc deprotection is carried out in 20% pyridine in DMF; (n) TFA, Tips, H2O, DODT; (o) GSSG, DMSO, Phosphate Buffer, HPLC purified.

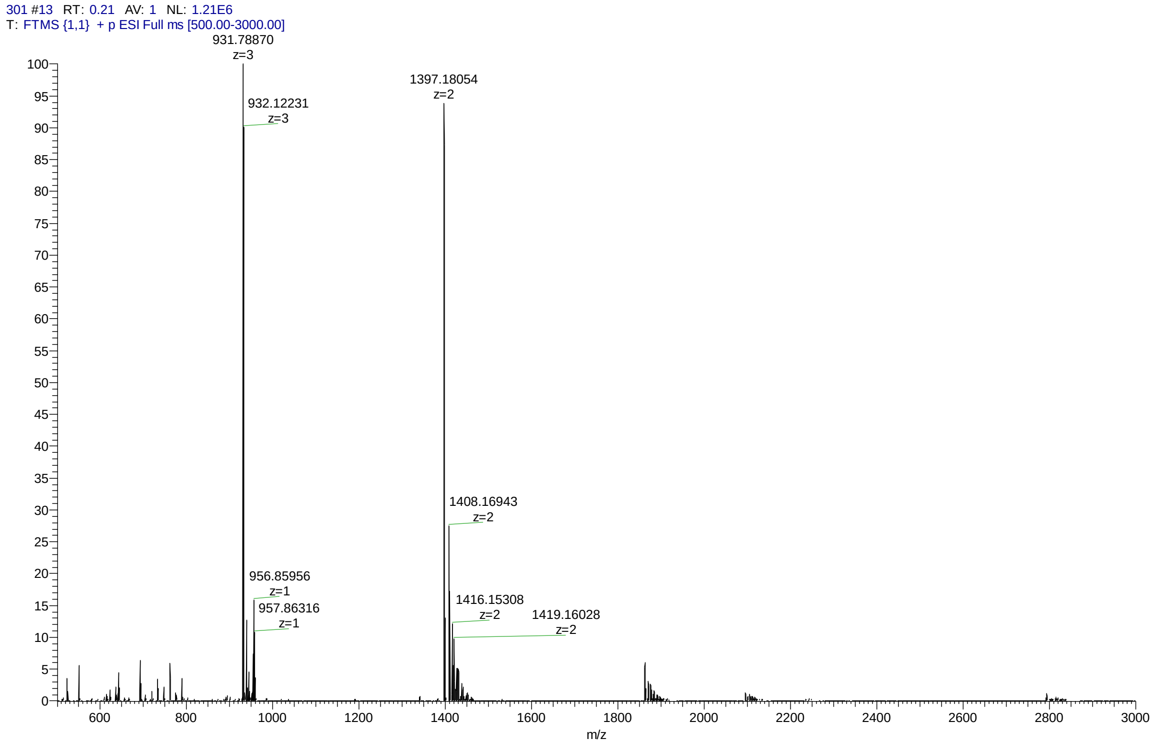
301 #13 RT: 0.21 AV: 1 NL: 1.21E6
T: FTMS {1,1} + p ESI Full ms [500.00-3000.00]
931.78870 z=3
932.12231 z=3
1397.18054 z=2
1408.16943 z=2
956.85956 z=1
957.86316 z=1
1416.15308 z=2
1419.16028 z=2
m/z

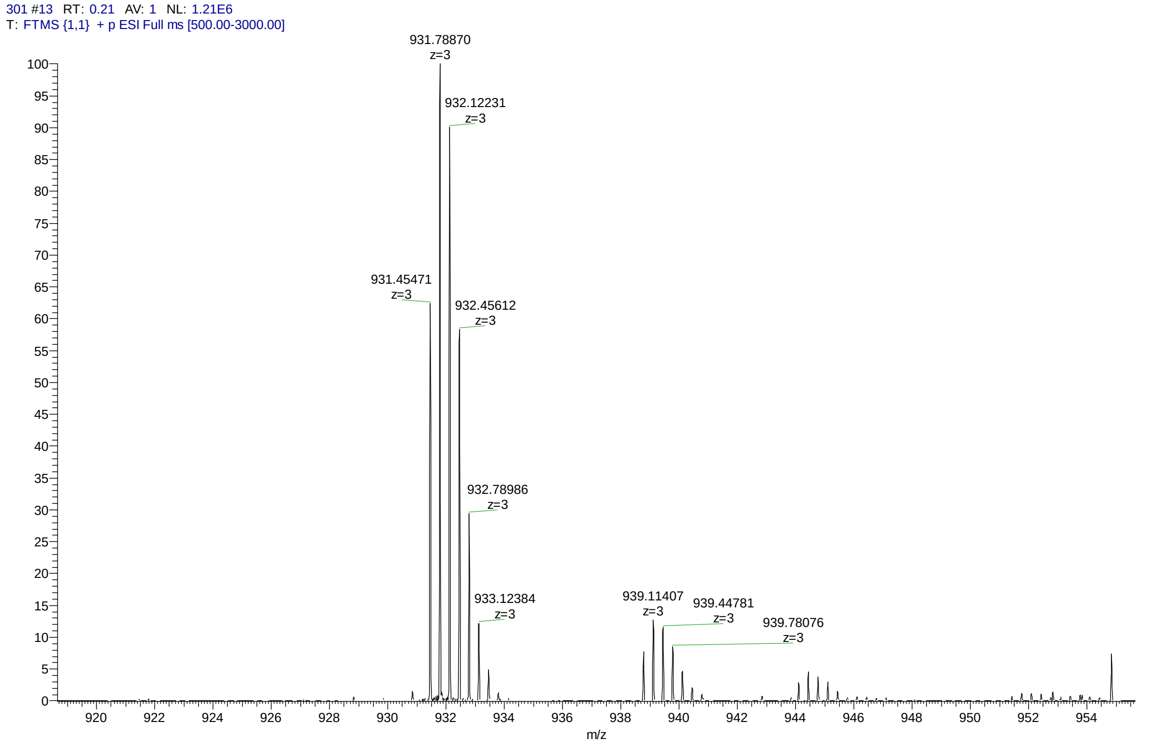
301 #13 RT: 0.21 AV: 1 NL: 1.21E6
T: FTMS {1,1} + p ESI Full ms [500.00-3000.00]
931.78870 z=3
932.12231 z=3
931.45471 z=3
932.45612 z=3
932.78986 z=3
933.12384 z=3
939.11407 z=3
939.44781 z=3
939.78076 z=3
m/z

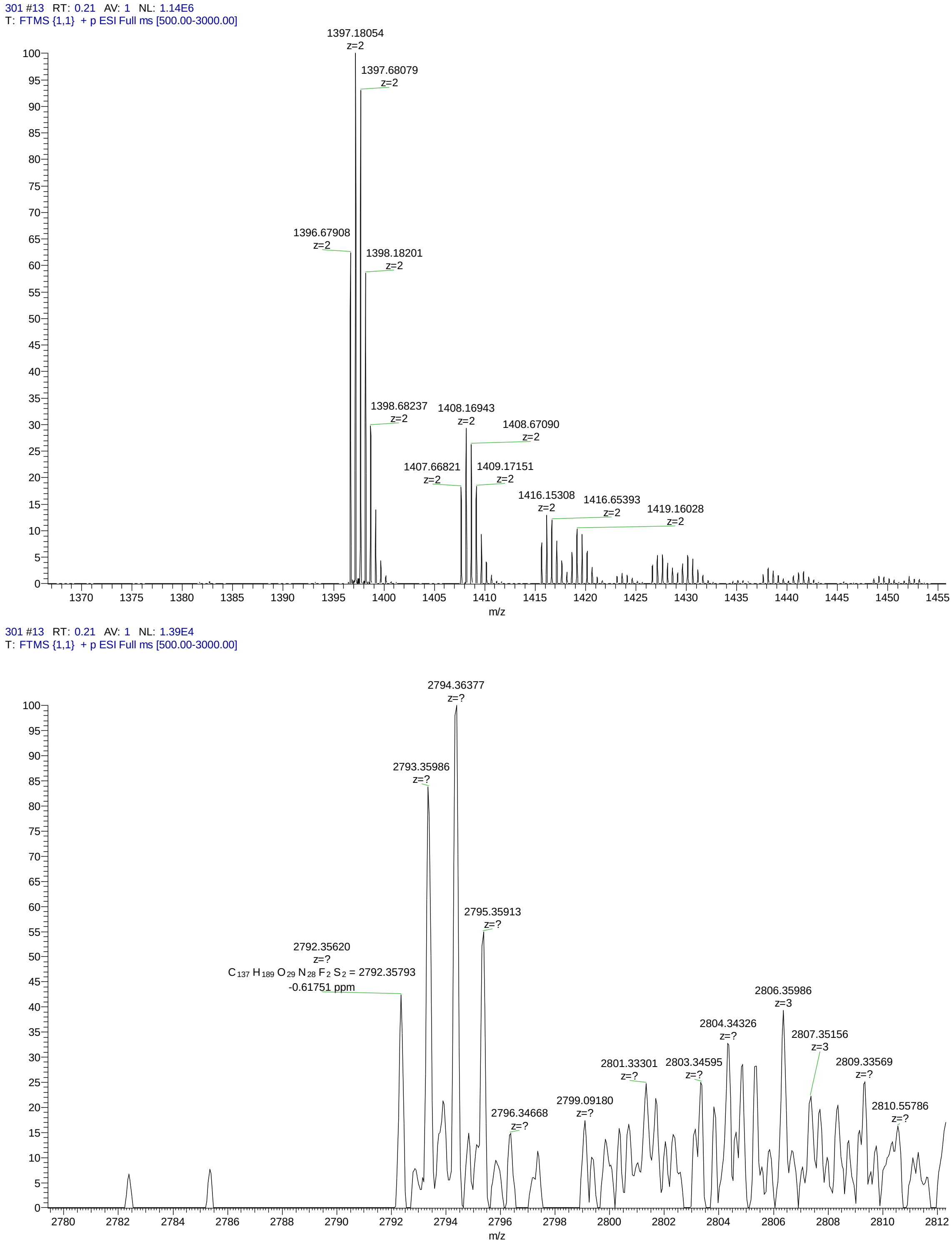

Figure S1. Chemical Synthesis and Mass spectra of TATE-46.

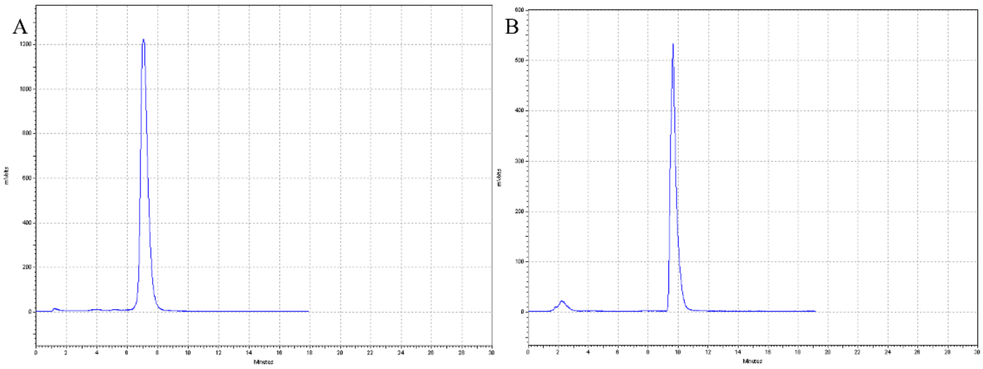

Figure S2. Analytic radio-HPLC chromatograms of ^{68}Ga-DOTA-TATE(A) and ^{68}Ga-FAPI-46(B).

Table S1. Biodistribution results of ^{68}Ga-TATE-46 in NCI-H727 tumor-bearing mice at 0.5, 1, 2, and 4 hours p.i.

Organs / Time	0.5	1	2	4
Blood	13.53±2.03	11.27±2.53	6.13±0.61	4.09±0.21
Brain	0.37±0.06	0.36±0.03	0.23±0.04	0.24±0.02
Heart	4.71±0.30	4.15±0.83	2.41±0.35	1.90±0.09
Lung	7.03±1.58	5.51±0.97	3.67±0.68	2.69±0.17
Liver	10.02±2.51	10.52±2.84	8.40±0.12	8.08±0.87
Spleen	4.48±0.72	4.01±0.79	3.05±0.38	3.04±0.09
Kidney	23.86±8.92	37.00±3.89	26.46±4.77	22.40±6.23
Stomach	6.84±0.73	6.68±0.95	6.18±0.43	5.05±0.01
Intestine	4.58±2.16	3.74±0.72	2.85±1.12	1.82±1.11
Bone	4.84±0.25	4.80±1.08	4.65±0.87	4.49±0.06
Muscle	2.40±0.48	2.39±0.49	2.36±0.14	2.36±0.32
**Tumor**	3.56±0.69	7.53±1.13	7.08±0.88	6.48±1.82

(Note: each mouse received 5.55-7.4 MBq. Data are presented as mean ± SD, n=3)

Table S2. Biodistribution of ^{68}Ga-TATE-46, ^{68}Ga-DOTA-TATE, and ^{68}Ga-FAPI-46 in NCI-H727 tumor-bearing mice at 1h p.i.

Organs / probes	^{68}Ga-TATE-46	^{68}Ga-DOTA-TATE	^{68}Ga-FAPI-46
Blood	11.27±2.53	0.42±0.15	1.05±0.05
Brain	0.36±0.03	0.03±0.01	0.05±0.01
Heart	4.15±0.83	0.23±0.09	0.39±0.16
Lung	5.51±0.97	5.22±0.82	3.17±0.79
Liver	10.52±2.84	1.35±0.28	2.56±0.95
Spleen	4.01±0.79	1.22±0.11	3.75±0.75
Kidney	37.00±3.89	9.18±3.65	2.25±0.12
Stomach	6.68±0.95	7.98±0.89	0.38±0.01
Intestine	3.74±0.72	1.48±0.11	0.38±0.01
Bone	4.80±1.08	0.19±0.03	0.51±0.05
Muscle	2.39±0.49	0.09±0.05	0.18±0.02
Tumor	7.53±1.13	1.57±0.17	1.07±0.01

(Note: each mouse received 5.55-7.4 MBq. Data are presented as mean ± SD, n=3; $*p<0.05$, $**p<0.01$, $***p<0.001$.)

Immunohistochemistry and immunofluorescence

1. Main equipment

Name	Manufacturer	Type
Dewatering machine	Changzhou Zhongwei Electronic Instrument Co., Ltd	TSJ-II

Embedding machine	Changzhou Suburb Zhongwei Electronic Instrument Factory	BMJ-III
Paraffin cutting machine	Shanghai Leica Instrument Co., Ltd	RM2016
Blade	Japanese feather safety razor Co., Ltd	R35
Glass slides	Nantong Meiweide Experimental Equipment Co., Ltd	P105-2001
Cover glass	Nantong Meiweide Experimental Equipment Co., Ltd	CS01-2450
Drying oven	Shanghai Jinghong Experimental Equipment Co., Ltd	DHG-9148A
Repair instrument	Thermo Shandon Limited	A80400011
Bleaching shaker	Shanghai Chengjie Instrument Equipment Co., Ltd	HY-5
Vortex Mixer	Jiangsu Xinkang Medical Device Co., Ltd	XK80-A
Pocket centrifuge	USA SCLOGEX	S1010E
Pipetting gun	Dalong Xingchuang Experimental Instrument (Beijing) Co., Ltd. (DragonLab)	7010101004/7010101006/ 7010101014 / 7010101014
Grouping pen	Beijing Lanjieke Technology Co., Ltd	BC004

2. Main reagents

Name	Manufacturer	Type	Dilution ratio
Anhydrous ethanol	Chemical Reagent Co., Ltd	1000292680	75%、85%、95%
Dewaxing liquid	Wuxi Jiangyuan Industrial Technology and Trade Co., Ltd	240131	/
Citrate buffer solution (dry powder)	Servicebio	GA2307051	1:1000
PBS buffer (dry powder)	Servicebio	G0002-2L	1:1000
Hydrogen peroxide	Servicebio	G1204	0.5%
Bovine serum albumin (BSA)	Servicebio	GC305010	3%
DAB kit	Beijing Zhongshan Jinqiao Biological Co., Ltd	ZL1-9018	1:20
Hematoxylin dye solution	Beijing Bailingwei Technology Co., Ltd	LM10N13	Working fluid
Hematoxylin differentiation liquid	Servicebio	G1039	Working fluid
Hematoxylin return blue liquid	Servicebio	G1040	Working fluid
Fap primary antibody (fap)	abcam	ab314456	1:100
Antibody (sstr2)	abcam	ab134152	1:100
Secondary antibody (HRP-labeled goat anti-rabbit))	Servicebio	GB22303	1:100

3. Immunohistochemistry experimental methods

(1) Paraffin sections were dewaxed to water: the sections were successively placed in dewaxing solution Ⅰ 15min, dewaxing solution Ⅱ 15min, dewaxing solution Ⅲ 15min, and anhydrous ethanol Ⅰ 5min; Anhydrous ethanol Ⅱ 5min, 85% alcohol 5min, 75% alcohol 5min, distilled water.

(2) Antigen repair: Dip the section into the repair solution, microwave oven repair for 20min; After cooling, wash 3 times with PBS for 5min each time.

(3) Blocking endogenous peroxidase: the slices were placed in 3% hydrogen peroxide, incubated at room temperature away from light for 25min, and the slides were placed in PBS and washed three times on a decolorizing shaking table for 5min each time.

(4) Serum blocking: add bovine serum (BSA) at room temperature for 20min.

(5) Add the prepared primary antibody and place the slices horizontally in a wet box at 4°C for overnight incubation.

(6) Wash with PBS for 3 times, 5min each time; Drop the second antibody, 37℃ 30 min; Wash with PBS for 3 times, 5min each time.
(7) DAB color development: Prepare fresh DAB color development solution, drop it onto the tissue, develop color at room temperature, control the color development time under the microscope, positive color is brown and yellow, and wash the slice with distilled water to terminate the color development.
(8) Restaining nucleus: hematoxylin restaining for 3min, wash with tap water, and rinse with running water after the water is returned to blue.
(9) Dehydrated sealing: the slices were immersed in 75%, 85%, 95%, anhydrous ethanol, xylene for 10min, respectively, and neutral gum sealing.

4. Immunofluorescence experimental methods
(1) Paraffin sections were dewaxed to water: the sections were successively put into dewaxing solution Ⅰ 15min, xylene Ⅱ 15min, xylene Ⅲ 15min, anhydrous ethanol Ⅰ 5min; Anhydrous ethanol Ⅱ 5min, 85% alcohol 5min, 75% alcohol 5min, distilled water.
(2) Antigen repair: Dip the section into the repair solution, microwave oven repair, repair for 20min; After cooling, wash 3 times with PBS for 5min each time.
(3) Blocking endogenous peroxidase: the slices were placed in 3% hydrogen peroxide, incubated at room temperature away from light for 25min, and the slides were placed in PBS and washed three times on a decolorizing shaking table for 5min each time.
(4) Serum blocking: bovine serum (BSA), incubated at room temperature for more than 30 minutes.
(5) Add the first primary antibody: overnight at 4℃, wash with PBS for 3 times, 5min each time. The slices were placed flat in a wet box at 4°C and incubated overnight.
(6) The corresponding HRP labeled secondary antibody (HRP labeled goat anti-rabbit) was added and incubated at room temperature for 50min; Wash with PBS 3 times, 5min each time.
(7) Amplification of TSA signal: TSA (FITC-Tyramide) was added and incubated at room temperature for 10min away from light; After incubation, wash 3 times in PBS for 5min each time.
(8) Antigen repair: Dip the section into the repair solution, microwave oven repair for 10min, cease fire for 8min, and then repair for 10min; After cooling, wash 3 times with PBS for 5min each time.
(9) Add the second antibody: add the prepared primary antibody, and the slices are placed flat in a wet box at 4°C and incubated overnight; Wash with PBS for 3 times, 5min each time.
(10) Add the second antibody: add the second antibody corresponding to the second antibody and incubate at 37℃ for 30 min; Wash with PBS for 3 times, 5min each time.
(11) DAPI restaining nuclei: DAPI was added and incubated at room temperature for 10min; Wash with PBS for 3 times, 5min each time.
(12) Sealing tablets: anti-fluorescence quenching sealing tablets.
